# MicroRNA132 Modulates Short-Term Synaptic Plasticity but Not Basal Release Probability in Hippocampal Neurons

**DOI:** 10.1371/journal.pone.0015182

**Published:** 2010-12-29

**Authors:** Talley J. Lambert, Daniel R. Storm, Jane M. Sullivan

**Affiliations:** 1 Graduate Program in Neurobiology and Behavior, University of Washington, Seattle, Washington, United States of America; 2 Department of Pharmacology, University of Washington, Seattle, Washington, United States of America; 3 Department of Physiology and Biophysics, University of Washington, Seattle, Washington, United States of America; INSERM U901, France

## Abstract

MicroRNAs play important regulatory roles in a broad range of cellular processes including neuronal morphology and long-term synaptic plasticity. MicroRNA-132 (miR132) is a CREB-regulated miRNA that is induced by neuronal activity and neurotrophins, and plays a role in regulating neuronal morphology and cellular excitability. Little is known about the effects of miR132 expression on synaptic function. Here we show that overexpression of miR132 increases the paired-pulse ratio and decreases synaptic depression in cultured mouse hippocampal neurons without affecting the initial probability of neurotransmitter release, the calcium sensitivity of release, the amplitude of excitatory postsynaptic currents or the size of the readily releasable pool of synaptic vesicles. These findings are the first to demonstrate that microRNAs can regulate short-term plasticity in neurons.

## Introduction

While extensive research has explored the role of miRNAs in processes like development and pathogenesis, the function of miRNAs in the adult nervous system is only just beginning to emerge. With their ability to regulate the proteomic composition of neuronal compartments, it stands to reason that miRNAs might play a role in shaping the functional properties of neurons. miRNAs and their precursors are present in synaptic fractions along with components of the miRNA machinery [1], [2] where together they are poised to regulate neurotransmission.

MicroRNA-132 (miR132) is a highly conserved miRNA that is induced by the neurotrophin BDNF in a CREB-dependent manner [3]. In culture, upregulation of miR132 increases dendritic outgrowth in an activity-dependent fashion via suppression of a GTPase-activating protein [3], [4]. miR132 has also been shown to regulate cellular excitability in cultured cells, possibly via regulation of ion channels [5]. We have recently demonstrated that miR132 is rapidly transcribed in the hippocampus *in vivo* following enhanced neuronal activity and contextual fear conditioning [6]. Exposure to light also induces transcription of miR132 in the SCN *in vivo*, where it plays a role in regulating entrainment of the circadian clock [5].

Because miR132 has been found to affect neuronal morphology and excitability, we investigated the effects of miR132 overexpression on synaptic transmission and short-term plasticity. Here we show that overexpression of miR132 increases the paired-pulse ratio and decreases synaptic depression without affecting initial presynaptic release probability or postsynaptic sensitivity to neurotransmitter. These data indicate that miR132 selectively influences short-term plasticity without altering basal synaptic transmission.

## Results

To achieve overexpression of miR132, we engineered replication-deficient Lentivirus to encode the primary transcript of miR132 (pri-miR132). To identify infected neurons, these vectors also expressed enhanced Green Fluorescent Protein (EGFP) as a separate reporter protein driven by an internal ribosome entry site (IRES). Control neurons were infected with a Lentivirus that only contained the IRES-EGFP reporter sequence. To control for the specificity of miR132, in some experiments we also infected neurons with a Lentivirus encoding the primary transcript of miR1, a non-neural, heart specific miRNA. Overexpression of the mature miR132 transcript was verified by quantitative real-time PCR. Averaged over 3 biological replicates, miR132-overexpressing neurons had a roughly 45-fold increase in the amount of mature miR132 transcript relative to EGFP-infected and miR1-infected controls at the age of recording (13–15 days in culture; fold increase of miR132 transcript relative to EGFP: miR132 47.3±7.7, *n* = 3; miR1 1.2±0.3, *n* = 2).

### Overexpression of miR132 enhanced paired-pulse facilitation

**Figure 1:**
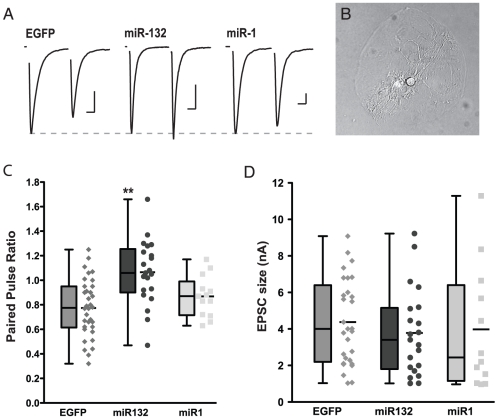
Overexpression of miR132 increases short-term facilitation without affecting basal release probability. (A) Representative traces showing typical paired-pulse responses from EGFP-, mir132-, and miR1-overexpressing neurons with stimulus artifacts and presynaptic action currents blanked for clarity. Traces have been normalized to the first response in the pair to compare facilitation. (scale bars: 1nA, 10ms) (B) DIC image of a representative autaptic neuron growing in isolation on a microisland of permissive substrate. (C) The PPR was greater in neurons overexpressing miR132 relative to EGFP and miR1 control neurons (miR132 PPR was 1.4 times EGFP PPR and 1.2 times miR1 PPR; **, *P*<0.005). (D) EPSC size was not significantly different between miR132-overexpressing and EGFP and miR1 control groups. Box plots represent quartiles, and whiskers show min and max values, while adjacent scatter plots show individual data points with mean.

To study the effects of miR132 overexpression on synaptic transmission, we monitored the paired-pulse ratio (PPR; Figure 1A), a measure that is commonly used to identify changes in presynaptic release probability [7], in autaptic hippocampal neurons (Figure 1B). The PPR was calculated by delivering two stimuli 50 msec apart and then dividing the amplitude of the second excitatory postsynaptic current (EPSC) by the amplitude of the first EPSC. Whereas both EGFP and miR1 control neurons demonstrated paired-pulse depression (i.e. the second response was smaller than the first), expression of miR132 led to paired-pulse facilitation (i.e. the second response was larger than the first) and a significant change in the PPR (Figure 1C; EGFP 0.77±0.04, *n* = 34; miR132 1.06±0.06, *n* = 21; miR1 0.87±0.05, *n* = 13; One way ANOVA, *P*<0.0005). These results show that miR132 influences short-term plasticity, perhaps by modulating release probability.

### Overexpression of miR132 decreased synaptic depression

**Figure 2:**
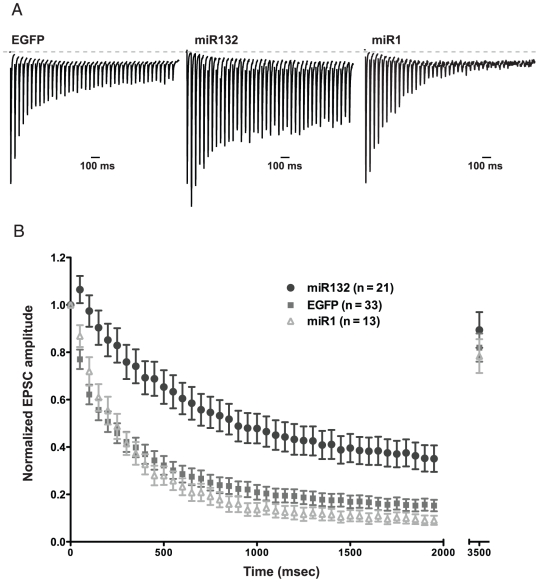
Overexpression of miR132 decreases synaptic depression in response to a train of stimuli, but does not affect the rate of refilling of the RRP. (A) Representative traces showing responses to a train of 40 stimuli delivered at 20 Hz. Data were collected at a rate of one train every 60 s to allow full recovery between trains. Traces have been normalized to the amplitude of the first stimulus in the train to facilitate comparison of synaptic depression. (B) The peak amplitude of each EPSC in the train was normalized to the peak amplitude of the first EPSC and plotted versus time to show the rate of depression during the train. The amplitude of a single EPSC evoked 1.5 s after the end of the train was used to measure recovery from depletion of the pool of readily releasable vesicles. Neurons overexpressing miR132 demonstrate significantly reduced synaptic depression during the train relative to EGFP and miR1-overexpressing controls at all time points (*P*<0.05), but no change in recovery after the train.

We next stimulated neurons with a 40-pulse train at 20 Hz and assessed the rate of synaptic depression during the train by normalizing the amplitude of each EPSC to the amplitude of the first EPSC in the train. We found a pronounced decrease in the rate of depression throughout the train in miR132 expressing neurons, relative to EGFP and miR1 controls (Figure 2B; 10^th^ Stimulus: EGFP 0.34±0.04, *n* = 33; miR132 0.69±0.07, *n* = 21; miR1 0.28±0.04, *n* = 13; One way ANOVA, *P*<0.0001; 40^th^ Stimulus: EGFP 0.15±0.02, *n* = 33; miR132 0.35±0.06, *n* = 21; miR1 0.09±0.02, *n* = 13; One way ANOVA, *P*<0.0005).

Like the fast synchronous component of release, the slower asynchronous component also tended to be greater at the end of the train in miR132 overexpressing neurons. The ratio of the ratio of total asynchronous to synchronous release during a train in miR132 overexpressing neurons, while reduced, was not significantly different from EGFP controls (EGFP 2.32+/− 0.27, *n* = 21; miR132 1.66+/− 0.25, *n* = 20; *P*>0.05). These data suggest that miR132 modulates a mechanism that influences the short-term plasticity of both synchronous and asynchronous neurotransmission, although the effect on synchronous release appears to be greater.

miR132-mediated reduction in synaptic depression might be caused by an increase in the rate of refilling of the readily releasable pool of synaptic vesicles (RRP), leading to a larger steady-state synaptic response at the end of the train. However, when the rate of refilling of the RRP was monitored using the response to a test stimulus delivered 1.5 s following the RRP-depleting train, there was no difference in the extent of recovery in neurons overexpressing miR132 (Figure 2B; EGFP 0.82±0.06, *n* = 33; miR132 0.89±0.08, *n* = 21; miR1 0.78±0.07, *n* = 13; One way ANOVA, *P*>0.5). Thus, the reduction in synaptic depression seen in miR132-overexpressing neurons in response to either pairs of stimuli or a longer train is not likely due to an increase in the rate of refilling of the RRP.

### Overexpression of miR132 did not affect basal synaptic transmission

The increase in PPR and the reduction of synaptic depression seen in miR132 expressing neurons could be consistent with a reduction in basal release probability [7]. However, we found no effect of miR132 or miR1 on EPSC size (Figure 1D; EGFP 4.37±0.43 nA, *n* = 21; miR132 3.78±0.52 nA, *n* = 21; miR1 3.98±0.97 nA, *n* = 21; One way ANOVA, *P*>0.7). Because a change in release probability would be expected to change EPSC size, this lack of effect suggests that the overexpression of miR132 does not influence basal release probability.

### Overexpression of miR132 did not affect mEPSC size or frequency

**Figure 3:**
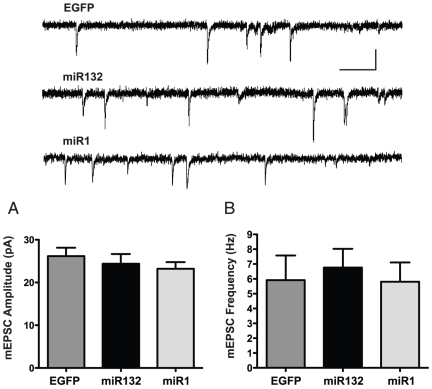
miR132 does not affect the size or the frequency of spontaneous mEPSCs. (*Top*) Traces show typical spontaneous mEPSCs recorded from control and miR132-overexpressing neurons (Scale bars, 20 pA, 100 ms). (A) There was no difference between groups in the average amplitude of mEPSCs. (B) There was no difference between groups in the average frequency of mEPSCs. (for all groups, *n* = 15).

In order to detect possible changes in postsynaptic sensitivity to neurotransmitter or in the number of functional synapses, we tested the effect of miR132 overexpression on spontaneous miniature EPSCs (mEPSCs). Figure 3 shows that there was no change in either the size (EGFP 26.2±2.0 pA; miR132 24.4±2.3 pA; miR1 23.21±1.6 pA; all groups *n* = 15; One way ANOVA, *P*>0.5), or frequency (EGFP 5.9±1.7 Hz; miR132 6.8±1.3 Hz; miR1 5.8±1.3 Hz; all groups *n* = 15; One way ANOVA *P*>0.8) of mEPSCs in miR132-overexpressing neurons relative to EGFP- and miR1-overexpressing control neurons. These findings suggest that the overexpression of miR132 does not affect the number of functional excitatory synapses or postsynaptic sensitivity to glutamate in hippocampal neurons.

### Overexpression of miR132 did not affect the size of the RRP or *P*_VR_

**Figure 4:**
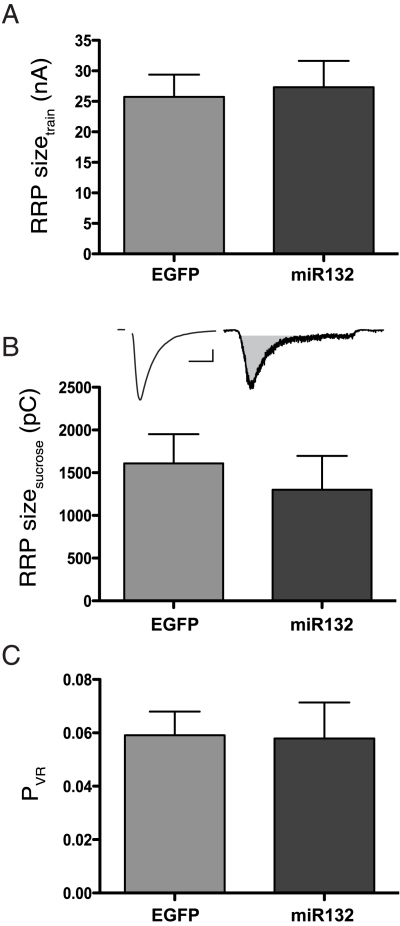
Decreased synaptic depression in miR132-overexpressing neurons is not due to a change in the size of the readily releasable pool or vesicular release probability. (A) The average RRP size was not different between miR132-overexpressing neurons and EGFP control neurons using the cumulative amplitude method. (both groups *n* = 21) (B) There was no difference between groups in RRP size estimated by hypertonic challenge. Representative traces from an individual cell demonstrate a single evoked EPSC (left) and the response recorded during hypertonic challenge (right). Gray area represents charge integral used as the measurement of the RRP (scale bar: 1nA, 1ms and 400pA, 1s for EPSC and sucrose trace respectively; both groups *n* = 8). (C) There was no difference between groups in the vesicular release probability (charge integral of the EPSC divided by the charge integral of the RRP; both groups *n* = 8).

Depression during a train is commonly interpreted to represent depletion of the RRP [8], [9], [10]. To estimate the size of the RRP, we plotted the cumulative EPSC amplitude during the train, calculated the linear regression fit to the last 8 stimuli, and used the Y-intercept of this line at time *t* = 0 to estimate the cumulative amplitude at rest [8], [11]. Using this method, we found no difference between the sizes of the RRP in control versus miR132-overexpressing neurons (Figure 4A; EGFP 25.7±3.6 nA, *n* = 21; miR132 27.3±4.3 nA, *n* = 21; *P*>0.75). The result was the same when we used the cumulative charge method instead of cumulative EPSC amplitude (data not shown) [11].

To confirm these results with a calcium-insensitive protocol, we used hypertonic challenge to release all immediately available synaptic vesicles and estimate RRP size (see Experimental Methods). Again, we saw no difference in the size of the RRP between groups when measured with this method (Figure 4B; EGFP 1609±341 pC, *n* = 8; miR132 1301±396 pC, *n* = 8; *P*>0.55). In addition, these experiments allowed us to directly determine whether the vesicular probability of release (*P*_VR_) was altered, by calculating the fraction of the RRP released in response to a single action potential, measured as the average charge integral of the EPSC divided by the charge integral of the non-steady state sucrose response. Our analyses revealed no significant difference in *P*_VR_ between miR132 overexpressing neurons and EGFP controls (*P*_VR:_ EGFP 0.06±0.01, *n* = 8; miR132 0.06±0.01, *n* = 8; *P*>0.90).

### Calcium dependence of release is not altered by miR132 overexpression

**Figure 5:**
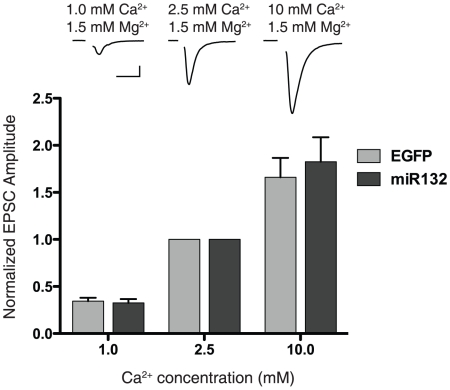
Calcium dependence of release is not altered by overexpression of miR132. External calcium was varied while extracellular magnesium concentration remained constant at 1.5 mM. EPSC peak amplitudes measured in 1 mM and 10 mM were normalized to the values measured in 2.5 mM calcium. miR132 overexpression had no significant effect on the calcium dependence of release (for all groups, *n* = 9, t-test for 1 mM and 10 mM *P*>0.05; scale bar for representative traces: 1nA, 10 ms).

Given that miR132 has been linked with dendritic outgrowth, a decrease in calcium sensitivity of release concomitant with an increase in synapse number in miR132 overexpressing neurons could conceivably explain the results observed in the present study. To rule out the possibility that overexpression of miR132 modifies the calcium dependence of release, we varied the external calcium concentration and measured the corresponding changes in EPSC peak amplitudes (normalized to EPSCs in the standard 2.5 mM external calcium) in miR132 and EGFP infected neurons. The normalized peak EPSC amplitudes were not significantly different between groups at either 1 mM or 10 mM external calcium (Figure 5; 1 mM Ca^2+^: EGFP 0.35±0.04, miR132 0.33±0.04, *P*>0.70; 10 mM Ca^2+^: EGFP 1.66±0.21, miR132 1.83±0.26, *P*>0.60; *n* = 9 for all groups). Therefore, our data suggest that miR132 does not affect the calcium dependence of release.

### Overexpression of miR132 did not affect postsynaptic receptor desensitization

Recently, it has been demonstrated that lateral diffusion of postsynaptic AMPA-type glutamate receptors (AMPARs) and subsequent exchange of desensitized receptors for naïve ones can contribute to recuperation from synaptic depression on millisecond timescales [12]. It is conceivable that a molecular target of miR132 negatively regulates processes such as postsynaptic receptor mobility or recovery from postsynaptic AMPAR desensitization. To test this directly, we measured PPR before and after application of cyclothiazide (CTZ) to block AMPAR desensitization. While CTZ led to a slight (non-significant) decrease in the PPR in both EGFP controls and neurons overexpressing miR132, there was no difference in the magnitude of the effect of CTZ on the PPR of these two groups (PPRctz/PPRctrl: EGFP 0.83±0.09, *n* = 8; miR132 0.83±0.06, *n* = 8; *P*>0.99), indicating that miR132 does not enhance facilitation through postsynaptic effects on AMPAR mobility or desensitization.

## Discussion

MicroRNA-132 is a neurotrophin-induced miRNA that has been demonstrated to affect neuronal characteristics such as neurite outgrowth and cell excitability. Because of its documented ability to regulate cellular characteristics in an activity dependent manner [3], [4], [5], [6], a role for miR132 in synaptic function was investigated. In the present study, we provide evidence that overexpression of miR132 in cultured hippocampal neurons leads to selective changes in short-term synaptic plasticity. Specifically, we observed an increase in the paired-pulse ratio and a decrease in the amount of synaptic depression in response to a train of stimuli. This phenotype was not accompanied by any evidence for changes in presynaptic vesicular release probability, nor was it caused by changes in the size or the rate of refilling of the readily releasable pool. Furthermore, it cannot be explained by miR132-induced changes in the calcium sensitivity of release or postsynaptic receptor desensitization.

While changes in short-term plasticity are often correlated with changes in vesicular release probability, the dissociation of these two measurements observed in the present study is not without precedent. Synaptic depression is often considered a combination of reduced presynaptic glutamate release and altered properties of postsynaptic AMPARs following glutamate binding [7]. In addition, upregulation of neuronal calcium sensor 1 (NCS-1) has been shown to enhance neurotransmitter release during pairs of stimuli and stimulus trains without altering basal release probability [13].

A gene ontology search of all the computationally predicted targets listed for miR132 in the mouse genome revealed the intriguing possibility that miR132 may negatively regulate the mRNA of the pore forming α_1A_ subunit of the P/Q-type calcium channel (Ca_v_2.1). Interestingly, a recent paper [14] demonstrated increased paired-pulse facilitation and decreased synaptic depression with acute selective pharmacological blockade of Ca_v_2.1, an identical phenotype to that shown here. While a reduction of P/Q-type calcium channels would also be predicted to decrease the probability of release, it is possible that levels of N-type calcium channels are increased in compensation, countering any change in basal release probability and yielding results similar to Scheuber et al. This possibility is supported by the fact that N-type channels are upregulated in Ca_v_2.1 KO mice [15]. A strong caveat to this candidate mechanism, however, comes from Ishikawa and colleagues [16] who, using the aforementioned Ca_v_2.1 KO mice to compare the basic properties of N-type and P/Q-type calcium currents in the calyx of Held, found that P/Q-type, but not N-type, calcium currents undergo activity-dependent facilitation [16], in conflict with the results of Scheuber et al. [14]. Thus, while Ca_v_2.1 is an intriguing computationally predicted target of miR132, its regulation by miR132 remains to be demonstrated and the expected phenotypic effect of such regulation is debated.

The fact that we observed no change in either EPSC size or the frequency of spontaneous mEPSCs suggests that there was no change in the number of synapses made by miR132-overexpressing neurons. This result was slightly unexpected given that upregulation of miR132 was previously reported to increase neurite outgrowth [3], [4]. Indeed, two very recent studies did observe increases in spontaneous mEPSC frequency following either overexpression of miR132 [17] or downregulation of p250GAP, a target of miR132 [18]. Together, these results suggest that the effects of miR132 on spontaneous release and neuronal morphology are complex, and likely influenced by undetermined variables.

Increasingly, research shows that miRNAs play a role in regulating synaptic formation, maturation, and function [19]. A growing body of evidence also implicates miRNAs in the regulation of long-term plasticity in the mature nervous system [20], [21], [22]. However, to our knowledge, no previous study has demonstrated a role for miRNAs in short-term synaptic plasticity. Our findings suggest that miR132 can modulate the computational properties of hippocampal neurons by regulating short-term plasticity in ways that promote facilitation and/or reduce synaptic depression without affecting basal synaptic transmission. The molecular mechanism of this effect remains elusive, and future studies will be aimed at evaluating candidate mRNA targets of miRNA132 that might mediate this phenotype.

## Methods

### Ethics Statement

Experiments were performed in accordance with University of Washington's guidelines for the care and use of animals and were approved by the Institutional Animal Care and Use Committee at the University of Washington. In addition, all experiments were performed in accordance with the University of Washington Environmental Health and Safety approved protocols.

### Neuronal Cultures

Neurons isolated from the hippocampi of P0–P1 wild-type (C57BL/6;129SvJ) mice were cultured on small microislands of permissive substrate as previously described [23]. Neurons were plated onto a feeder layer of astrocytes laid down 1–7 days earlier, then grown without mitotic inhibitors and used for recordings at 13–15 days in culture.

### Viral Constructs

At 2–3 days in culture, hippocampal neurons were infected with Lentivirus encoding one of the following: 1) the miR132 primary transcript along with EGFP as a real-time visual reporter of infection, 2) miR1 primary transcript along with EGFP as a control for nonspecific microRNA overexpression, or 3) EGFP alone as a control for viral infection and EGFP expression. Lenti virions were generated according to the Invitrogen ViraPower Lentiviral Expression System manual using the Lenti plasmid pRRL-cPPT-CMV-X-PRE-SIN [24]. Expression of mature miR132 was verified by quantitative real-time PCR using the TaqMan miRNA assay from Applied Biosystems, Inc. (Foster City, CA).

### Electrophysiology

Whole-cell voltage-clamp recordings were obtained from isolated autaptic neurons after 13–15 days in microisland culture using an Axopatch 200B amplifier (Molecular Devices, Sunnyvale, CA). All experiments included measurements obtained from two or more different culture preparations, and within each preparation were always performed on age-matched neurons. The standard extracellular solution contained 119 mM NaCl, 5 mM KCl, 2.5 mM CaCl_2_, 1.5 mM MgCl_2_, 30 mM glucose, 20 mM Hepes, and 1 µM glycine. Recording pipettes of 2–5 MΩ were filled with 148.5 mM potassium gluconate, 9 mM NaCl, 1 mM MgCl2, 10 mM Hepes, and 0.2 mM EGTA; the chloride concentration in these solutions facilitates the distinction between excitatory (glutamatergic) and inhibitory (GABAergic) autapses.

To elicit EPSCS, the membrane potential was held at −60 mV, and synaptic responses were evoked by triggering unclamped “action currents” with a 1-ms depolarizing step to +40 mV. The size of the recorded responses was calculated as peak amplitude. Series resistance was monitored, and only cells with stable series resistance were included in the data analysis. Series resistance was compensated 75–85% to reduce errors in measuring large currents. While there are likely some residual errors in our peak current measurements, these are expected to be comparable between all groups—given the lack of effect of miR132, miR1 and EGFP expression on EPSC size—and are therefore not responsible for the effects on short-term plasticity observed with miR132 expression. Furthermore, post-hoc analysis showed that membrane resistance, membrane capacitance, and series resistance did not differ between groups (R_m_: EGFP 111.5±16.8 MΩ, *n* = 26; miR132 116.8±15.8 MΩ, *n* = 21, *P*>0.80; C_m_: EGFP 42.7±2.6 pF, *n* = 27; miR132 40.6±4.0 pF, *n* = 11, *P*>0.65; R_S_: EGFP 9.0±0.7 MΩ, *n* = 27; miR132 7.7±0.6 MΩ, *n* = 11, *P*>0.19).

For the repetitive stimulation protocol, a 20 Hz train of 40 stimuli (1 msec steps from −60 mV to +40 mV) was delivered every 60 sec, and each EPSC in the train was normalized to the peak amplitude of the first EPSC. To measure recovery from depression, an additional test stimulus was delivered 1.5 sec after the end of the train. The size of the readily releasable pool was estimated using the protocol described previously [8], [11].

To assess the Ca^2+^ dependence of release, the [Ca^2+^] of the external recording solution was varied while extracellular magnesium concentration remained constant at 1.5 mM. EPSC peak amplitudes measured in 1 and 10 mM [Ca^2+^]_e_ were normalized to the values measured in 2.5 mM [Ca^2+^]_e_. The 2.5 mM [Ca^2+^]_e_ values were acquired before and after solution changes and then averaged to control for rundown. Solutions were exchanged with a gravity-feed bath perfusion system. Immediately after achieving whole-cell configuration, spontaneous mEPSCs were recorded continuously over 10 sec periods. Peak amplitudes of spontaneous mEPSCs were measured offline semi-automatically by using an adjustable amplitude threshold. All deflections from baseline greater than threshold were detected. Selected events were then visually examined, and any spurious events were manually rejected, and any missed events were flagged for inclusion in the mean amplitude and frequency calculations. mEPSC frequencies were calculated by dividing the total number of mEPSC events by the total time sampled.

The size of the readily releasable pool of vesicles was measured with hypertonic solution by applying divalent-free extracellular solution containing 0.5 M sucrose to an isolated autaptic neuron using a puffer pipette controlled by a picospritzer. A vacuum pipette was used to clear the hypertonic solution rapidly. The hypertonic solution was applied over the entire island on which the autaptic neuron was located. This solution evoked a large initial transient current that declined to a low steady-state level over about 3 sec; hypertonic solution was applied for 4–5 sec in order to deplete the readily releasable pool fully. The size of the readily releasable pool was calculated by integrating the current evoked by the hypertonic solution to yield a charge value. To estimate the readily releasable pool size more accurately, we corrected the integral of the current by subtracting away the amount of steady-state exocytosis that occurred during the hypertonic solution flow (see Figure 4B).

### Data Analysis

Data were analyzed with 2-tailed unpaired t-tests with Welch's correction (equal variance not assumed) or with one way ANOVA as indicated. All data are presented as mean ± SEM.
